# Inhalation of marijuana affects *Drosophila* heart function

**DOI:** 10.1242/bio.044081

**Published:** 2019-07-19

**Authors:** Ivana M. Gómez, Maia A. Rodríguez, Manuela Santalla, George Kassis, Jorge E. Colman Lerner, J. Oswaldo Aranda, Daniela Sedán, Dario Andrinolo, Carlos A. Valverde, Paola Ferrero

**Affiliations:** 1 Centro de Investigaciones Cardiovasculares ‘Dr. Horacio E. Cingolani’, Facultad de Ciencias Médicas, UNLP, La Plata 1900, Argentina; 2 Universidad Nacional del Noroeste de la Provincia de Buenos Aires, Pergamino 2700, Argentina; 3 Medicalbit, La Plata 1900, Argentina; 4 Centro de Investigación y Desarrollo en Ciencias Aplicadas Facultad de Ciencias Exactas, CCT La Plata-UNLP-CICPBA, La Plata 1900, Argentina; 5 Programa Ambiental de Extensión Universitaria. Facultad de Ciencias Exactas–UNLP, La Plata 1900, Argentina; 6 Centro de Investigaciones del medio Ambiente Facultad de Ciencias Exactas, CCT La Plata-UNLP, La Plata 1900, Argentina

**Keywords:** *Drosophila*, Cannabis, Cardiac, THC, CBD, Calcium

## Abstract

We investigated the effect of inhalation of vaporized marijuana on cardiac function in *Drosophila melanogaster*, a suitable genetic model for studying human diseases. Adult flies were exposed to marijuana for variable time periods and the effects on cardiac function were studied. Short treatment protocol incremented heart-rate variability. Contractility was augmented only under prolonged exposure to cannabis and it was associated with incremented calcium transient within cardiomyocytes. Neither the activity of the major proteins responsible for calcium handling nor the calcium load of the sarcoplasmic reticulum were affected by the cannabis treatment. The observed changes manifested in the cardiomyocytes even in the absence of the canonical cannabinoid receptors described in mammals. Our results are the first evidence of the *in vivo* impact of phytocannabinoids in *D. melanogaster.* By providing a simple and affordable platform prior to mammalian models, this characterization of cardiac function under marijuana exposure opens new paths for conducting genetic screenings using vaporized compounds.

This article has an associated First Person interview with the first author of the paper.

## Abstract (precis)

**Summary:** Marijuana induces early arrhythmogenesis and increases contractility in *Drosophila.* Our approach using this animal model allows us to carry out screenings to explore the impact of cannabinoids.

## INTRODUCTION

The human body normally produces endocannabinoids, molecules that trigger receptor-mediated signaling pathways (Elphick and Egertova, 2001). The endocannabinoid system regulates physiological processes such as release of neurotransmitters, perception of pain and cardiovascular, gastrointestinal and hepatic functions. The pharmacological manipulation of endocannabinoid levels or administration of cannabinoid agonists such as those from *Cannabis sativa* has been used for the treatment of several pathologies. The marijuana plant produces more than 60 terpenophenolic cannabinoid compounds in its trichomes, which are present in leaves and flowers. The main cannabinoids are cannabidiol (CBD), tetrahydrocannabinol (THC) and cannabinol (CBN). A meta-analysis on the use of cannabinoids for therapeutic purposes provided evidence on their efficacy in pain control, antiemesis in chemotherapy, stimulation of appetite in HIV patients, modulation of sleep disorders, motor dysfunction in paraplegia and in the treatment of anxiety disorders, diabetes and metabolic syndrome, among others (Schrot and Hubbard, 2016). However, the study of side effects must be carefully evaluated. Smoking or inhaling marijuana has been associated with increased risk of infarct and angina in patients with heart diseases (Durst and Lotan, 2011). Nevertheless, we still lack an exhaustive analysis of cardiac performance after exposure to cannabinoids. Results arising from animal models are complex, according to experimental conditions and route of administration. In this context, the inhalation route is one of the least explored.

*Drosophila melanogaster* has been studied by geneticists for a long time. Their small size, innumerable gene-specific mutants available in large collections, advanced techniques for genomic, transcriptomic and proteomic studies, gene-editing and strategies for ectopic gene expression control turn this animal into a feasible model for research. In addition, a growing amount evidence reinforces its usefulness as a model for human diseases such as epilepsy (Kuebler et al., 2001), obesity (Diop and Bodmer, 2012), Alzheimer’s disease (Finelli et al., 2004), Parkinson’s disease (Penney et al., 2016; Xiong and Yu, 2018), diabetes (Hallier et al., 2016) and heart disease (Santalla et al., 2014; Wolf et al., 2006) among others. One point of interest is the study of compounds like ethanol, cocaine, methamphetamines and nicotine. Pioneering research using these substances has provided information about the relationships between behavior, genes and pathways, even conserved between *Drosophila* and mammals (Kaun et al., 2012).

Despite advances in the knowledge of the effects induced by these substances, the analysis of cardiac function in this model provides a novel field to test the role of different compounds in the corresponding pathophysiology, both in wild-type and mutant organisms. *Drosophila* holds a particular advantage for the study of cardiac phenotypes: the independence between the cardiac system and the mechanism of gas exchange (through the cuticle and the tracheal system). This allows the application of compounds that affect the heart without interfering with the availability of oxygen for survival.

It has been demonstrated that the fruit fly presents endocannabinoids in the hemolymph (Khaliullina et al., 2015). In invertebrates, endocannabinoids are fundamentally associated with immunological mechanisms (Salzet and Stefano, 2002). However, the presence of CB1 and CB2 or of any other mammalian cannabinoid receptors has not been reported in *D. melanogaster* (McPartland et al., 2001; Salzet and Stefano, 2002). Herein, we test the effects of vaporization of marijuana on the cardiac performance of *D. melanogaster* to obtain an initial overview and reference for future screenings.

## RESULTS AND DISCUSSION

Canonical receptors for endocannabinoids and phytocannabinoids such as those present in mammals are absent in the *Drosophila* genome (McPartland et al., 2006a,b). However, the fruit fly possesses its own endogenous cannabinoids (Khaliullina et al., 2015). Therefore, we hypothesized that this organism may be responsive to phytocannabinoids.

**Fig. 1.**
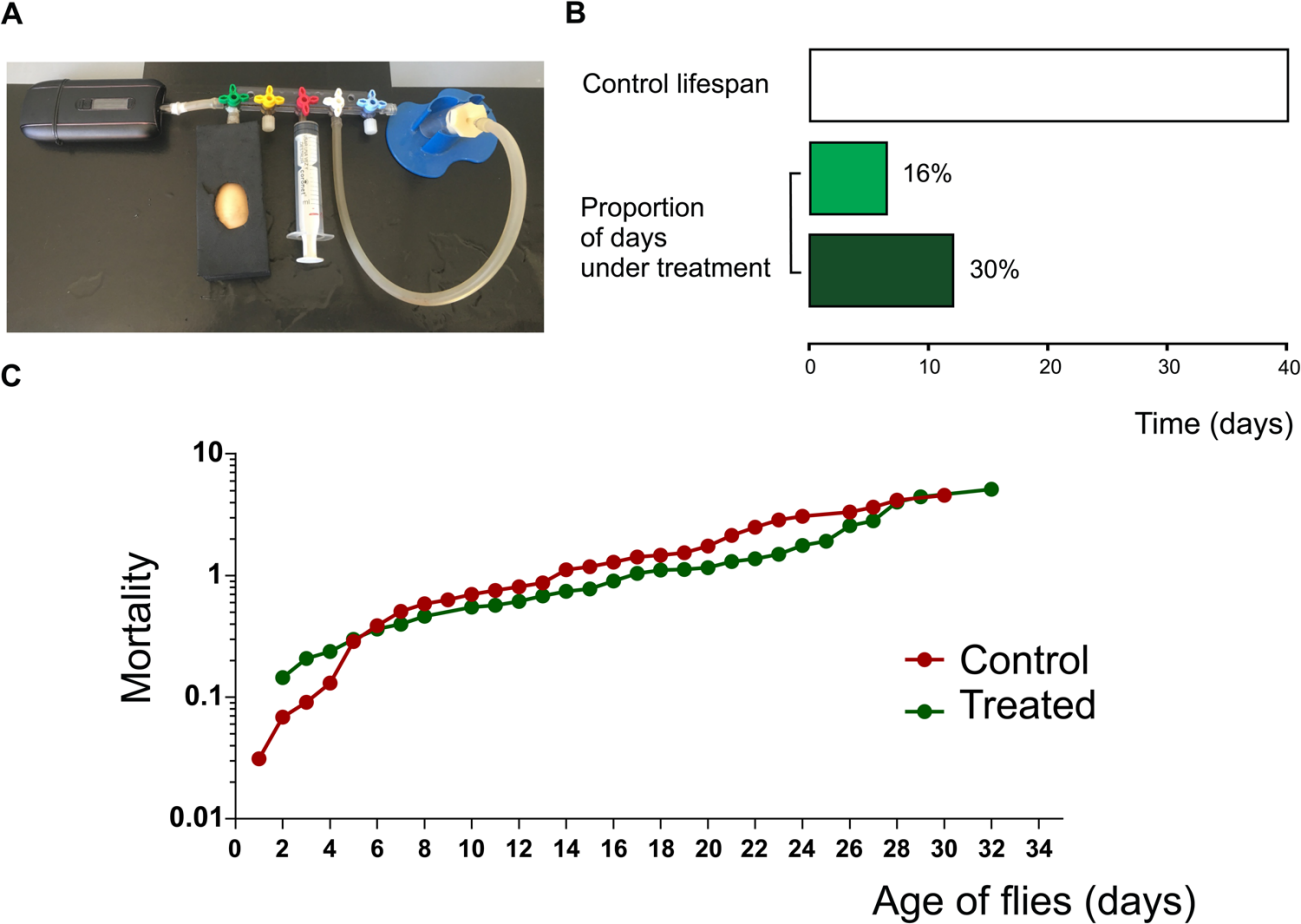
**Chronic exposure of *D. melanogaster* to vaporized herbal *Cannabis* sp. does not affect lifespan.** (A) Device connected to a vaporizer designed for providing air or marijuana into a vial containing flies. (B) Representation of the lifespan percentage of flies under treatment. (C) Mortality of treated (green) and non-treated (red) adult flies was represented throughout the life of two synchronized populations of adult flies that emerged from puparium. Air: *N*=196, treated: *N*=170. Statistical comparison and curves were obtained using the Kaplan–Meier method and analyzed using the log-rank (Mantel-Cox) test.

We developed a system for administrating vaporized substances to intact *Drosophila* flies. This method has been successfully extended to other models related to diseases and the consumption of substances, such as tobacco (Balcazar et al., 2017). Plant material was provided by a local NGO (www.mamacultiva.org), and consists of a cannabis strain that is currently used in the treatment of patients with refractory epilepsy. First, we determined the proportion of THC, CBD and CBN by gas chromatography coupled to mass spectrometry (Table S1). The marijuana strain used in the present study is rich in THC (114: 1 THC/CBD). Herbal cannabis was placed into a commercial vaporizer (Ascent by DaVinci™) connected to a custom device as shown in Fig. 1A. Temperature was set to 188°C (370°F). At this temperature, most cannabinoids were present in the air (Table S2). By controlling a three-way stopcock manifold, vapor was suctioned with the aid of a syringe and transferred into a vial containing the flies to be treated. Temperature inside the vial receiving the air coming from the vaporizer was measured with a temperature probe in order to verify that the vial's temperature (room temperature) did not change during exposition (data not shown). Flies were exposed to the resulting air containing cannabinoid compounds, which were incorporated through and distributed by the tracheal ramifications of the flies, eventually reaching the hemolymph. Although it is a passive process, we defined it as inhalation.

Survival and mortality studies were carried out to test if chronic administration of cannabis is a feasible approach to analyze heart function. In this aspect, consumption of cannabinoids along the entire life did not affect lifespan of flies (Fig. 1C).

**Fig. 2.**
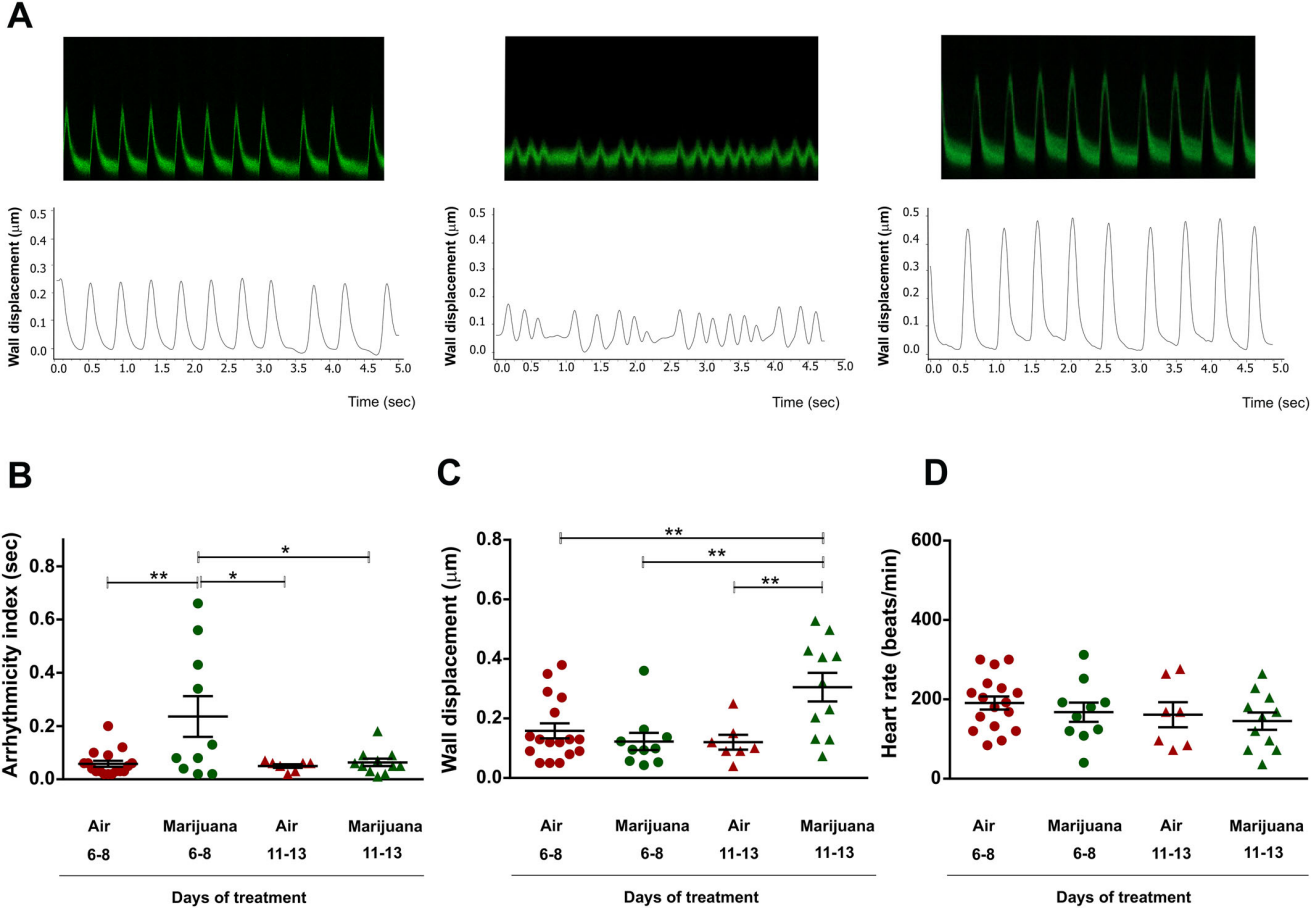
**Cardiac performance is modified by *Cannabis* sp.** Flies harboring the reporter system *hand*C-GFP (*hand*C-GFP/+) were analyzed. (A) Typical recordings (top) and digitalized signals (bottom) of heart wall displacements for each group during 5 s. (B–D) Average results (±s.e.m.). (B) Arrhythmicity index is incremented during short-term exposure to marijuana. (C) Contractility is significantly incremented in flies treated with marijuana for 11–13 days. (D) Heart rate remains unchanged. *N* = air 6–8 days (17), marijuana 6–8 days (10), air 11–13 days (7), marijuana 11–13 days (11). One-way analysis of variance (ANOVA) followed by Tukey's post hoc test. *P<0.05, **P<0.01.

Cardiac function was assessed in semi-intact heart preparations as we have previously described (Santalla et al., 2014; Santalla et al., 2016). A reporter system that expresses the green fluorescent protein (GFP) in cardiomyocytes and pericardial cells was used to track the movement of the individual cells during each cardiac cycle (systole and diastole). Individuals resulting from the cross between Canton-S and the homozygous *hand*C-GFP strain harbored one copy of the reporter system, enough to follow the movement of the heart wall. Mechanical parameters were defined as previously described (Balcazar et al., 2018). Fig. 2A shows representative mechanical recordings of cardiac performance.

With the aim to assess the cardiac performance, flies that emerged from the puparium were assigned to different groups: individuals exposed to marijuana for 6–8 days and individuals exposed for 11–13 days. Other groups of flies received only ambient air (control) for the same periods of time as the treated flies. Cannabis treatment applied for 6–8 or 11–13 days correspond to 13% or 30%, respectively, of the flies' average total lifespan (Fig. 1B). Therefore, we consider these periods of exposure to cannabis as chronic treatments.

Short exposure to cannabis (6–8 days) induced an increase in arrhythmicity index (Fig. 2B), which indicates that this phenomenon is an early and transitory effect because it was not observed in individuals exposed to the drug for 11–13 days. An early increment of arrhythmicity index may be expected because heart performance should adapt to a stimulation induced by the compounds. This arrhythmic pattern might be responsible for the early incremented mortality in the treated flies compared to non-exposed individuals (Fig. 1C). Flies under the short period of treatment (5 days), but analyzed 5 days after interruption of cannabis exposure, did not exhibit heart-rate variability (Fig. S1A). In all groups, inhaled marijuana did not modify the heart rate as a function of the exposure time (Fig. 2D and Fig. S1C). Studies on patients show that exposure to smoked marijuana induces abnormalities on cardiac rhythm (Akins and Awdeh, 1981; Fisher et al., 2005; Johnson and Domino, 1971; Kosior et al., 2001; Rezkalla and Kloner, 2018; Singh, 2000). Early arrhythmogenesis is observed, for example, only during the onset of reperfusion in mammal heart after a period of ischemia (Vittone et al., 2002). It is feasible that arrhythmias of the *Drosophila* heart in response to cannabis treatment represents one of the early consequences of adaptive mechanisms that the heart employs for reaching an equilibrium or steady state under cannabis consumption.

We then explored the effect of different inhalation times on cardiac contractility (inotropism) evaluated by assessing cardiac wall displacement. Fig. 2C shows that wall displacement increased significantly after prolonged inhalation of *Cannabis* sp. (11–13 days), but not in response to short-term treatment (6–8 days). Moreover, heart wall displacement was not incremented in flies exposed to ambient air and also not in a group of flies exposed for a short period (5 days) and then grown for an additional 5 days after treatment interruption (Fig. S1B). Taken together, these results suggest that the increase in contractility observed in the 11–13 days-treated flies is a late effect induced by prolonged exposure to marijuana.

Calcium signaling is essential for the cardiac contraction (systole) and relaxation (diastole) that occur when electrical activity is followed by a mechanical action in the cardiomyocytes. The link between the electrical and mechanical activity is known as excitation-contraction coupling (ECC), which depends on the intracellular Ca^2+^ concentration (Ca^2+^ transient). When an action potential depolarizes the cell membrane, the intracellular Ca^2+^ concentration increases due to a massive Ca^2+^ release from the sarcoplasmic reticulum (SR) into the cytosol, where Ca^2+^ binds to the myofilaments, thus inducing its contraction. For relaxation to occur, Ca^2+^ is quickly removed from the cytosol mainly by the SR Ca^2+^ATPase pump (SERCA) and the Na^+^/ Ca^2+^ exchanger (NCX). Slow removal systems like mitochondrial Ca^2+^ transporter and the sarcolemmal Ca^2+^ATPase pump (PMCA) contribute to a lesser extent to the reduction of Ca^2+^_i_ (Desai-Shah et al., 2010). These mechanisms govern cardiac contractility in *Drosophila* and mammals (Bers, 2001). Therefore, we studied whether mishandling of Ca^2+^ is involved in the cardiac phenotypes induced by the exposure to marijuana.

**Fig. 3.**
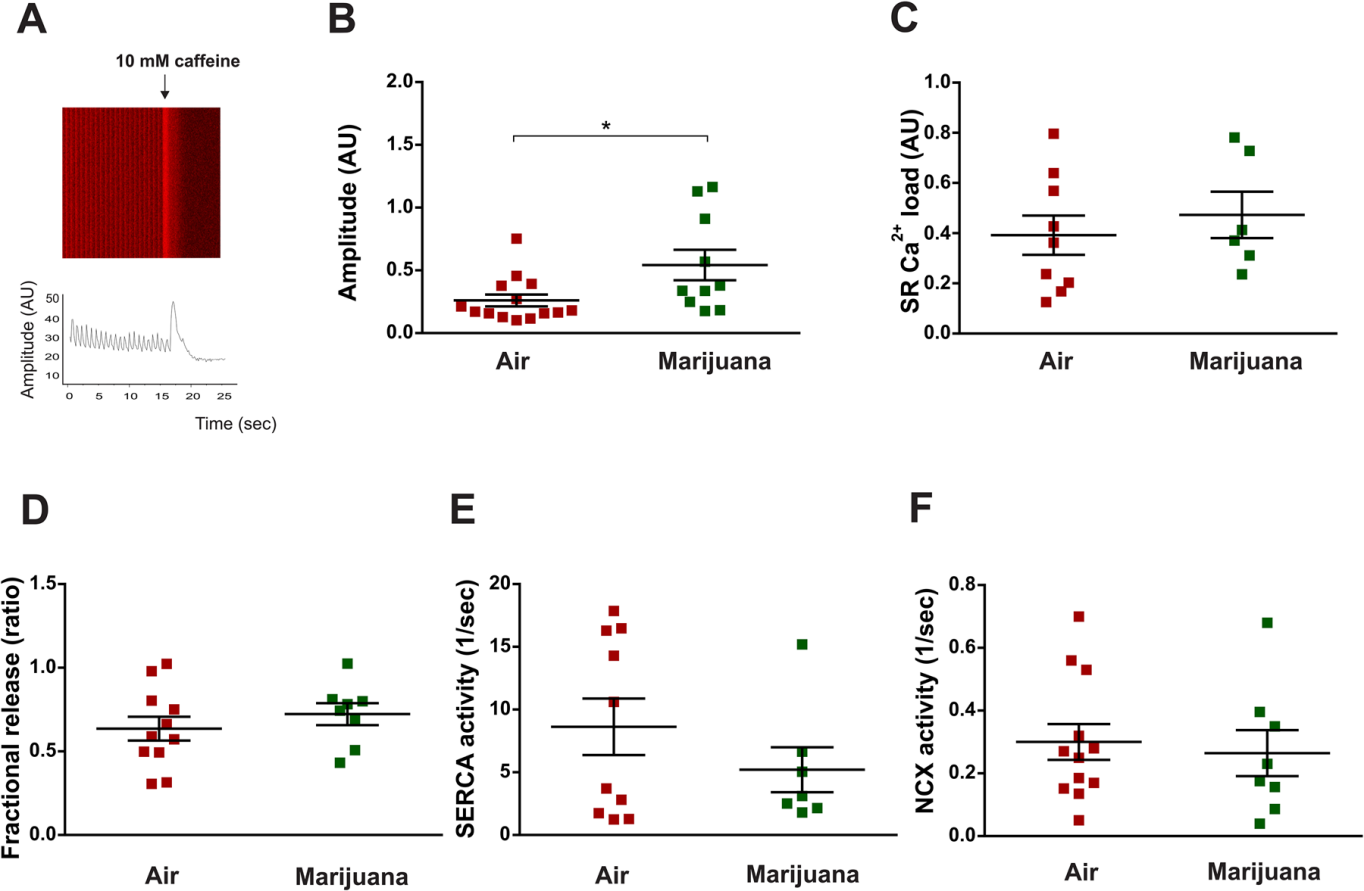
**Cannabis increments Ca^2+^ transient amplitude but does not modify SR calcium load, fractional release, SERCA and NCX activity in the *Drosophila* heart.** Ten-day-old flies harboring the reporter system GCaMP3 (genotype: UAS-GCaMP3/+; tinCΔ4-Gal4, UAS-GCaMP3/+) were exposed to air or marijuana. (A) Image and representative tracing of Ca^2+^ transient. A pulse of 10 mM caffeine is applied to the semi-intact preparation and visualized as a sudden increase in the fluorescence signal. (B–F) Average results (±s.e.m.). (B) Amplitude of Ca^2+^ transient [*N* = air (15), marijuana (10)]. (C) SR Ca^2+^ content [*N* = air (9), marijuana (6)]. (D) Fractional release [*N* = air (11), marijuana (7)]. (E) SERCA activity [*N* = air (12), marijuana (8)]. (F) NCX activity [*N* = air (12), marijuana (8)]. Student's *t*-test (two-tailed) was utilized for comparison between two groups. *P<0.05.

Ten-day-old flies bearing one copy of the genetically-encoded Ca^2+^ reporter (GCaMP3) in the heart, were vaporized with cannabis or ambient air (control). Cytosolic Ca^2+^ transients were assessed and SR Ca^2+^ content was estimated by application of a 10 mM caffeine pulse applied onto the semi-intact heart preparation (Fig. 3A). This pharmacological intervention, widely used in mammalian studies, opens the SR Ca^2+^ channels (ryanodine receptors, RyR) allowing SR- Ca^2+^ release. The amplitude of caffeine-induced Ca^2+^ transient estimates the SR- Ca^2+^ content. Comparison between pre-caffeine and caffeine-induced Ca^2+^ transients' decay, allows calculating the relative importance of each rapid removal system during relaxation. The results obtained from the analyses of the flies that consumed cannabis were compared with those obtained from flies which received air only.

We observed an increment in cytosolic Ca^2+^ transients in marijuana-treated flies (Fig. 3B), which was associated to incremented contractility in response to prolonged exposure to marijuana (Fig. 2C). Unexpectedly, the observed increment in the Ca^2+^ transient cannot be attributed to an increase in either SR Ca^2+^ load or fractional Ca^2+^ release because these two parameters were not significantly modified by marijuana inhalation (Fig. 3C,D). Moreover, estimated activity of the main proteins involved in Ca^2+^ handling remain unchanged (Fig. 3E,F). The reason for these somewhat unexpected results is not apparent to us. A possible explanation relates to myofilaments and Ca^2+^ interaction. Myoﬁlament calcium sensitivity results from the balance between Ca^2+^ association and dissociation rates (k_on_ and k_off_) with the components of troponin/tropomyosin complex. This complex modulates actin/myosin interaction and therefore the contractile force produced by the cardiac muscle (Chung et al., 2016). In this context, possible reduced association rates and/or incremented dissociation rates could be contributing to an increase in cytosolic Ca^2+^ transient. Marijuana inhalation might produce a simultaneous decrease in myofilament Ca^2+^ sensitivity, which would increase the intracellular Ca^2+^ transient – and consequently an increment in the activity of cross-bridges – which would be responsible for the observed increase in cardiac shortening. Another possibility to be considered is that marijuana produces a prolongation of the action potential that allows a higher Ca^2+^ influx that can be extruded from the cell through the NCX. Further experiments are needed to explore these possibilities. Altogether, effects of chronic cannabis exposure on heart performance can be categorized as follows: 1, early changes like heart-rate variability; and 2, alterations caused by prolonged exposure to cannabis like the increment in *Drosophila* heart contractility (Fig. S2).

Finally, one additional set of experiments was carried out to test whether cannabis treatment induced relevant alterations of behavior, e.g. movement behavior, that could affect the heart performance of *Drosophila*. Three images were captured from video recordings comparing flies treated with air or marijuana. To assess individual activities, we counted the number of individuals in the top and the bottom half of vials utilized for treatments. We observed that flies exposed to cannabis crowded more frequently close to the top of the vials than flies that received air (Fig. S3). Of note, experiments using CP-55,940 – a synthetic cannabinoid – showed restored negative geotaxis activity (i.e. climbing capability) of flies that were exposed to paraquat (PQ) toxicity via a receptor-independent mechanism. Antioxidant activity of CP-55,940 and preservation of the dopaminergic pathway could be the cause of these effects (Jimenez-Del-Rio et al., 2008). In our conditions, we observed that flies exposed to marijuana crowed at the top of the vial. Although the treated group exhibited a significant difference compared to air-subjected individuals, we cannot deduce whether this behavior is due to cannabinoid-induced negative geotaxis or associated to alterations in flight muscle activity or other metabolic adjustments from the preference triggered by the odor accumulated in the top of the vial. This aspect should be studied more deeply.

Our findings demonstrate that cannabis affects cardiac function in *D. melanogaster*. However, we currently cannot discriminate between the beneficial or detrimental effects of cannabinoids. In view of our observations, short-term exposure appears to be deleterious, because arrhythmic patterns of heart activity manifested concomitant with an incremented mortality of flies. On the other hand, prolonged exposure to marijuana incremented contractility. An adaptive long-term mechanism might re-establish proper heart performance and this change could be interpreted as being beneficial. This appraisal is in concordance with the similarity in survival between non-treated and treated groups observed at this period of treatment (Fig. 1C).

Herein, we provide the first evidence of cardiac responsiveness to vaporized cannabinoids in *Drosophila*. These findings indicate that the fruit fly as a useful, low-cost and high-performance model for future tests of cardiac phenotypes induced by phytocannabinoids.

## MATERIALS AND METHODS

### *Drosophila* stocks, rearing and crosses

Fly stocks were maintained in vials at 28–29°C, partially filled with a mixture of maize flour, glucose, agar and yeast supplemented with 10% antimycotic. Assays were made with the wild-type strain *Ca*nton-S (BDSC# 9514) (Sharma et al., 2005) obtained from Bloomington Drosophila Stock Center. The *hand*C-GFP line, expressing the reporter protein GFP in heart and pericardial cells was used for contractility assessments. For cytosolic calcium sensing, we used flies harboring the reporter system GCaMP3 specifically expressed in heart cells (UAS-GCaMP3/UAS-G*Ca*MP3; tinCΔ4-Gal4, UAS-GCaMP3/tinCΔ4-Gal4, UAS-GCaMP3) (Lin et al., 2011). In flies, a minimal GAL4 activity is present at 16°C, while 29°C provides a balance between maximal GAL4 activity and minimal effects on fertility and viability when flies are grown at this temperature (Duffy, 2002). To obtain a significant fluorescence signal of this reporter, we utilized this temperature for growing the flies, including those harboring the *hand*C-GFP reporter system. *Hand*C-GFP flies were provided by Prof. Achim Paululat and G*Ca*MP3-expressing flies were provided by Dr Heiko Harten, both from University of Osnabrück, Germany (Sellin et al., 2006).

Wild-type (WT) flies were crossed with individuals carrying one of these reporters. We analyzed heterozygous individuals of F1 that possess one copy of the reporter system in order to obtain information on mechanical activity (using the *hand*C-GFP reporter) or Ca^2+^ transients (using the UAS-G*Ca*MP3 reporter).

### Characterization of cannabinoids components

For chemical characterization vegetal material was extracted with ethanol. An aliquot of the ethanolic extract was subjected to a clean-up treatment with C18, activated carbon and MgSO_4_ to adsorb impurities and contaminants. Clean extract was diluted in hexane and injected into a PerkinElmer Clarus 650 gas chromatograph, coupled to a PerkinlElmer Clarus SQ 8 S mass spectrometer. Determination of THC, CBD and CBN was conducted following the technique described by Tayyab and Shahwar (2015). The analytical standards of THC, CBD and CBN used were acquired from Cerilliant Corporation. The concentrations of THC, CBD and CBN (expressed in mg of cannabinoids/g of plant material) were determined in the cannabis strain used for the experiments (Table S1).

### Administration by vaporizing cannabis

A custom device (Fig. 1) was connected through one end to an Ascent Portable Vaporizer (DaVinci™) containing 0.03 g of dried herbal cannabis previously cut and carefully chopped. Temperature was set to 370°F (∼188°C), for vaporizing cannabinoids present in the plant tissue. The produced vapor was collected by a syringe, and 40 cc of vapor at room temperature was insufflated into a 15.5 ml plastic vial containing adult flies. The top end of the vial was equipped with a filter for avoiding increase in air pressure within the vial when vapor was insufflated. Vaporized individuals received two daily doses and remained in contact with the vaporized substances for 15 min each, before being transferred to their regular cultivation vials. Another set of experiments was designed to provide ambient air to the vials containing flies, the purpose of which was to corroborate that the cardiac effects are due to the vaporization of the cannabinoids and not caused by handling or temperature of the vapor.

Adults were exposed to ambient air or marijuana and classified in groups according to the duration of the treatment: 6–8 or 11–13 days. An addition group received the treatment for 5 days and individuals were grown for a further 5 days without receiving ambient air or cannabis. All groups were euthanized for assessing physiological cardiac parameters.

### Survival and mortality analysis

Populations of synchronized flies were separated in two groups. The first group received vaporized cannabis twice a day. The protocol was continued until the last individual died. The second group received ambient air. Survival analysis was carried out by counting live and dead flies every day. Daily mortality was estimated using this formula:
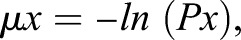


wherein *ln* is the natural logaritm of the daily survival rate, *ux* is the daily mortality rate and *Px* is the daily survival rate (Vermeulen and Bijlsma, 2004). Statistical comparison and curves were obtained using the Kaplan–Meier method and analyzed using the log-rank (Mantel-Cox) test.

### Evaluation of cardiac function in semi-intact heart preparations

Dissection of adult hearts was performed as previously described (Santalla et al., 2014). The procedure was performed under a Schonfeld Optik model XTD 217 stereomicroscope. Individuals were briefly anesthetized with carbon dioxide (CO_2_) and fixed by the dorsal region in a 60 mm Petri dish coated with a thin layer of petroleum jelly. The head and thorax were removed in this preparation and therefore neuronal influence on cardiac activity was minimal. The middle ventral region of the abdomen was opened and the internal organs were carefully removed. The preparation was submerged in oxygenated artificial hemolymph solution containing 5 mM KCl, 8 mM MgCl_2_, 2 mM CaCl_2_, 108 mM NaCl, 1 mM NaH_2_PO_4_, 5 mM HEPES,4 mM NaHCO_3_, 10 mM trehalose and 10 mM sucrose, pH 7.1. Calcium recordings and assessments of mechanical activity of these semi-intact heart preparations were carried out using Carl Zeiss LSM410 and LSM800 confocal microscopes.

### Mechanical activity and Ca^2+^ transient recording

Mechanical activity was recorded from all flies harboring the GFP protein under the control of the *hand*C driver expressed in cardiomyocytes and pericardial cells. A pericardial or a cardiac cell was focused with a 20× objective. We then tracked the fluorescent signal of a cardiac or pericardial cell edge from one side of the heart. We verified that the movement of the pericardial or cardiac cells is synchronic and that they displace to the same extent. The movement of a single cell expressing the GFP reporter was followed by laser scanning in line-scan mode by setting a line longitudinally to the displacement of either a pericardial cell or a cardiomyocyte. Recordings were obtained for 5 s. Cytosolic Ca^2+^ was assessed by the fluorescence produced by Ca*^2+^* binding to the GCaMP3 reporter. Semi-intact preparations were visualized using a 5× objective and the laser was focused to stimulate a minimal central region of the conical chamber where the intensity of signal was the highest. Recordings of 60 s allowed us to obtain a pattern of Ca^2+^ transients.

The images obtained were processed with a custom-made algorithm for Anaconda developed in our laboratory for obtaining the intensity of fluorescence from a pericardial cell/cardiomyocyte over a threshold value. The images were obtained sequentially in time and then converted into a digitalized image of cell displacement. After conversion of the image into a txt extension file containing fluorescence values and time, the files were analyzed with LabChart software (AD Instruments, CO, USA).

We measured the displacement of the heart wall, expressed in µm from the diastolic to the systolic positions of a cardiac or pericardial cell. Heart rate was obtained by counting the number of peaks and expressed as beats/min. We measured the heart period as the interval between two consecutive contraction peaks. Arrhythmicity index (AI) was calculated as the standard deviation of periods normalized by their average. For Ca^2+^ transient signal, we measured fluorescence intensity [(Fmax-F0)/F0)]. For estimating SR- Ca^2+^ load, fractional release, SERCA and NCX activities, a 10 mM caffeine pulse was applied to the semi-intact heart preparations of GCaMP3 flies using a micropipette. The amplitude of caffeine-induced Ca^2+^ transients represents an estimation of SR-Ca^2+^ load, while fractional release results from the ratio between pre-caffeine and caffeine-induced Ca^2+^ transients' amplitudes. Time of relaxation of pre-caffeine Ca^2+^ transient is determined by SERCA and NCX activity. Caffeine-induced Ca^2+^ transient relaxation is only determined by NCX and its activity was estimated as the inverse of the relaxation time constant (tau) of the caffeine-induced Ca^2+^ transient. SERCA activity was evaluated as the difference between the inverse of tau from pre-caffeine and caffeine-induced Ca^2+^ transients (1/tau pre-caff–1/tau caff).

Videos of vials containing flies without exposure or subjected to ambient air or marijuana were recorded and we select three captures (beginning, middle and end of each recording). Images of each video were analyzed using ImageJ software and flies localized on the top and bottom half were counted. We repeated this procedure at least for four doses in the treated groups. Percentage of flies on the top and bottom half of the vials were calculated as follows: (number of individuals in each region/number of total individuals)×100.

### Statistical analysis

One-way analysis of variance (ANOVA) followed by Tukey's post hoc test were utilized for comparison of the differences among three or more groups. Student's *t*-test (two-tailed) was utilized for comparison between two groups. Statistical comparison and curves were obtained using the Kaplan–Meier method and analyzed using the log-rank (Mantel-Cox) test. Proportion comparisons were made using Fisher test. A *P*-value <0.05 was considered significant.

## Supplementary Material

Supplementary information

## References

[BIO044081C1] Akins D. and Awdeh M. R. (1981). Marijuana and second-degree AV block. *South. Med. J.* 74, 371-373. 10.1097/00007611-198103000-00035 6261403

[BIO044081C2] Balcazar D., Paronzini-Hernández N., Santalla M., Valverde C. A., Mattiazzi A., Ferrero P. (2017). Tabaquism and cardiomyophathies. Effects of tobacco consumption on cardiac calcium handling in *Drosophila melanogaster*. *Medicine* 77, 1.

[BIO044081C3] Balcazar D., Regge V., Santalla M., Meyer H., Paululat A., Mattiazzi A. and Ferrero P. (2018). SERCA is critical to control the Bowditch effect in the heart. *Sci. Rep.* 8, 12447 10.1038/s41598-018-30638-9 30127403 PMC6102201

[BIO044081C4] Bers D. (2001). *Excitation-Contraction Coupling and Cardiac Contractile Force*. Kluwer Academic Publishers, Dordrecht.

[BIO044081C5] Chung J. H., Biesiadecki B. J., Ziolo M. T., Davis J. P. and Janssen P. M. (2016). Myofilament calcium sensitivity: role in regulation of in vivo cardiac contraction and relaxation. *Front. Physiol.* 7, 562 10.3389/fphys.2016.00562 28018228 PMC5159616

[BIO044081C6] Desai-Shah M., Papoy A. R., Ward M. and Cooper R. L. (2010). Roles of the sarcoplasmic/endoplasmic reticulum Ca2+-ATPase, plasma membrane Ca2+-ATPase and Na+/Ca2+ exchanger in regulation of heart rate in larval drosophila. *The Open Physiology Journal* 3, 16-36. 10.2174/1874360901003010016

[BIO044081C7] Diop S. B. and Bodmer R. (2012). Drosophila as a model to study the genetic mechanisms of obesity-associated heart dysfunction. *J. Cell. Mol. Med.* 16, 966-971. 10.1111/j.1582-4934.2012.01522.x 22303936 PMC3454526

[BIO044081C8] Duffy J. B. (2002). GAL4 system in Drosophila: a fly geneticist's Swiss army knife. *Genesis* 34, 1-15. 10.1002/gene.10150 12324939

[BIO044081C9] Durst R. and Lotan C. (2011). The potential for clinical use of cannabinoids in treatment of cardiovascular diseases. *Cardiovasc Ther.* 29, 17-22. 10.1111/j.1755-5922.2010.00233.x 20946323

[BIO044081C10] Elphick M. R. and Egertova M. (2001). The neurobiology and evolution of cannabinoid signalling. *Philos. Trans. R. Soc. Lond. B* 356, 381-408. 10.1098/rstb.2000.0787 11316486 PMC1088434

[BIO044081C11] Finelli A., Kelkar A., Song H.-J., Yang H. and Konsolaki M. (2004). A model for studying Alzheimer's Abeta42-induced toxicity in Drosophila melanogaster. *Mol. Cell. Neurosci.* 26, 365-375. 10.1016/j.mcn.2004.03.001 15234342

[BIO044081C12] Fisher B. A. C., Ghuran A., Vadamalai V. and Antonios T. F. (2005). Cardiovascular complications induced by cannabis smoking: a case report and review of the literature. *Emerg. Med. J.* 22, 679-680. 10.1136/emj.2004.014969 16113206 PMC1726916

[BIO044081C13] Hallier B., Schiemann R., Cordes E., Vitos-Faleato J., Walter S., Heinisch J. J., Malmendal A., Paululat A. and Meyer H. (2016). *Drosophila* neprilysins control insulin signaling and food intake via cleavage of regulatory peptides. *Elife* 5, 250 10.7554/eLife.19430 PMC5140268 27919317

[BIO044081C14] Jimenez-Del-Rio M., Daza-Restrepo A. and Velez-Pardo C. (2008). The cannabinoid CP55,940 prolongs survival and improves locomotor activity in *Drosophila melanogaster* against paraquat: implications in Parkinson's disease. *Neurosci. Res.* 61, 404-411. 10.1016/j.neures.2008.04.011 18538428

[BIO044081C15] Johnson S. and Domino E. F. (1971). Some cardiovascular effects of marihuana smoking in normal volunteers. *Clin. Pharmacol. Ther.* 12, 762-768. 10.1002/cpt1971125762 4936140

[BIO044081C16] Kaun K. R., Devineni A. V. and Heberlein U. (2012). *Drosophila melanogaster* as a model to study drug addiction. *Hum. Genet.* 131, 959-975. 10.1007/s00439-012-1146-6 22350798 PMC3351628

[BIO044081C17] Khaliullina H., Bilgin M., Sampaio J. L., Shevchenko A. and Eaton S. (2015). Endocannabinoids are conserved inhibitors of the Hedgehog pathway. *Proc. Natl. Acad. Sci. USA* 112, 3415-3420. 10.1073/pnas.1416463112 25733905 PMC4371992

[BIO044081C18] Kosior D. A., Filipiak K. J., Stolarz P. and Opolski G. (2001). Paroxysmal atrial fibrillation following marijuana intoxication: a two-case report of possible association. *Int. J. Cardiol.* 78, 183-184. 10.1016/S0167-5273(00)00459-9 11398765

[BIO044081C19] Kuebler D., Zhang H., Ren X. and Tanouye M. A. (2001). Genetic suppression of seizure susceptibility in *Drosophila*. *J. Neurophysiol.* 86, 1211-1225. 10.1152/jn.2001.86.3.1211 11535671

[BIO044081C20] Lin N., Badie N., Yu L., Abraham D., Cheng H., Bursac N., Rockman H. A. and Wolf M. J. (2011). A method to measure myocardial calcium handling in adult *Drosophila*. *Circ. Res.* 108, 1306-1315. 10.1161/CIRCRESAHA.110.238105 21493892 PMC3128985

[BIO044081C21] McPartland J., Di Marzo V., De Petrocellis L., Mercer A. and Glass M. (2001). Cannabinoid receptors are absent in insects. *J. Comp. Neurol.* 436, 423-429. 10.1002/cne.1078 11447587

[BIO044081C22] McPartland J. M., Agraval J., Gleeson D., Heasman K. and Glass M. (2006a). Cannabinoid receptors in invertebrates. *J. Evol. Biol.* 19, 366-373. 10.1111/j.1420-9101.2005.01028.x 16599912

[BIO044081C23] McPartland J. M., Matias I., Di Marzo V. and Glass M. (2006b). Evolutionary origins of the endocannabinoid system. *Gene* 370, 64-74. 10.1016/j.gene.2005.11.004 16434153

[BIO044081C24] Penney J., Tsurudome K., Liao E. H., Kauwe G., Gray L., Yanagiya A., R Calderon M., Sonenberg N. and Haghighi A. P. (2016). LRRK2 regulates retrograde synaptic compensation at the *Drosophila* neuromuscular junction. *Nat. Commun.* 7, 12188 10.1038/ncomms12188 27432119 PMC4960312

[BIO044081C25] Rezkalla S. and Kloner R. A. (2018). Cardiovascular effects of marijuana. *Trends Cardiovasc. Med*. 21, 452-455. 10.1016/j.tcm.2018.11.004 30447899

[BIO044081C26] Salzet M. and Stefano G. B. (2002). The endocannabinoid system in invertebrates. *Prostaglandins Leukot. Essent. Fatty Acids* 66, 353-361. 10.1054/plef.2001.0347 12052049

[BIO044081C27] Santalla M., Portiansky E. L. and Ferrero P. (2016). *Drosophila melanogaster*, an emerging animal model for the study of human cardiac diseases. *Rev. Argent Cardiol.* 84, 424-430.

[BIO044081C28] Santalla M., Valverde C. A., Harnichar E., Lacunza E., Aguilar-Fuentes J., Mattiazzi A. and Ferrero P. (2014). Aging and CaMKII alter intracellular Ca2+ transients and heart rhythm in *Drosophila melanogaster*. *PLoS ONE* 9, e101871 10.1371/journal.pone.0101871 25003749 PMC4087024

[BIO044081C29] Schrot R. J. and Hubbard J. R. (2016). Cannabinoids: medical implications. *Ann. Med.* 48, 128-141. 10.3109/07853890.2016.1145794 26912385

[BIO044081C30] Sellin J., Albrecht S., Kolsch V. and Paululat A. (2006). Dynamics of heart differentiation, visualized utilizing heart enhancer elements of the *Drosophila melanogaster* bHLH transcription factor Hand. *Gene Expr. Patterns* 6, 360-375. 10.1016/j.modgep.2005.09.012 16455308

[BIO044081C31] Sharma P., Asztalos Z., Ayyub C., de Bruyne M., Dornan A. J., Gomez-Hernandez A., Keane J., Killeen J., Kramer S., Madhavan M. et al. (2005). Isogenic autosomes to be applied in optimal screening for novel mutants with viable phenotypes in *Drosophila melanogaster*. *J. Neurogenet.* 19, 57-85. 10.1080/01677060591007155 16024440

[BIO044081C32] Singh G. K. (2000). Atrial fibrillation associated with marijuana use. *Pediatr. Cardiol.* 21, 284 10.1007/s002460010063 10818197

[BIO044081C33] Tayyab M. and Shahwar D. (2015). GCMS analysis of Cannabis sativa L. from four different areas of Pakistan. *Egy. J. Forensic Sci.* 5, 114-125. 10.1016/j.ejfs.2014.07.008

[BIO044081C34] Vermeulen C. J. and Bijlsma R. (2004). Changes in mortality patterns and temperature dependence of lifespan in *Drosophila melanogaster* caused by inbreeding. *Heredity* 92, 275-281. 10.1038/sj.hdy.6800412 14679396

[BIO044081C35] Vittone L., Mundina-Weilenmann C., Said M., Ferrero P. and Mattiazzi A. (2002). Time course and mechanisms of phosphorylation of phospholamban residues in ischemia-reperfused rat hearts. Dissociation of phospholamban phosphorylation pathways. *J. Mol. Cell. Cardiol.* 34, 39-50. 10.1006/jmcc.2001.1488 11812163

[BIO044081C36] Wolf M. J., Amrein H., Izatt J. A., Choma M. A., Reedy M. C. and Rockman H. A. (2006). *Drosophila* as a model for the identification of genes causing adult human heart disease. *Proc. Natl. Acad. Sci. USA* 103, 1394-1399. 10.1073/pnas.0507359103 16432241 PMC1360529

[BIO044081C37] Xiong Y. and Yu J. (2018). Modeling Parkinson's disease in *Drosophila*: what have we learned for dominant traits? *Front. Neurol.* 9, 228 10.3389/fneur.2018.00228 29686647 PMC5900015

