# Supplementary material for: Inhalation of marijuana affects Drosophila heart function
Source: Biol Open. 2019 Jul 19;8(8):bio044081. doi: 10.1242/bio.044081 (PMC6737967; doi:10.1242/bio.044081)
Supplement: Supplementary information [file biolopen-8-044081-s1.pdf]

Supplementary information

Strain/variety	Current informal name: strain Moby Dick	
Genetic	Hybrid that presents 60% genetic characteristic of *C. sativa subesp sativa* and 40% of *C. sativa subesp indica*. F1 from crossbreeding White Widow (*C. sativa subesp sativa* x *C. sativa subesp indica*) with Haze (*C. sativa*) strains.	
Chemical composition of cannabinoids	High content of THC.<br><br>CBD: 1.00 mg/g of sample<br>THC: 113.70 mg/g of sample	% THC+CBD+CBN<br><br>%CBD= 0.85%<br>%THC=96.6%
	CBN: 3.0 mg/g of sample<br>Ratio: THC/CBD= 114/1	%CBN=2.55%
Main medical use in Argentine	Refractory epilepsy	

Table S1. Genetic and chemical characterization of the studied strain of *Cannabis sp*. Vegetal material provided by the NGO "Mamá Cultiva" was analyzed for determining % content of THC, CBD and CBN.

Biology Open • Supplementary information

**Cannabinoids**	**Boiling point (in ºC)**
Deltha 9- THC	157
CBD	180
CBN	185
**Terpenoids**	
Beta myrcene	166-168
Beta caryophyllene	119
d-limonene	177
1,8-cineole	176
Alpha-pinene	156
Beta-cymene	177
Deltha-3-carene	168
**Flavonoids**	
Apigenin	178
Cannflavin A	182
Beta-sitosterol	134

Table S2. Vaporized components. Temperature at which the main components present in *Cannabis sp* are vaporized in our conditions using a device setting at 188°C.

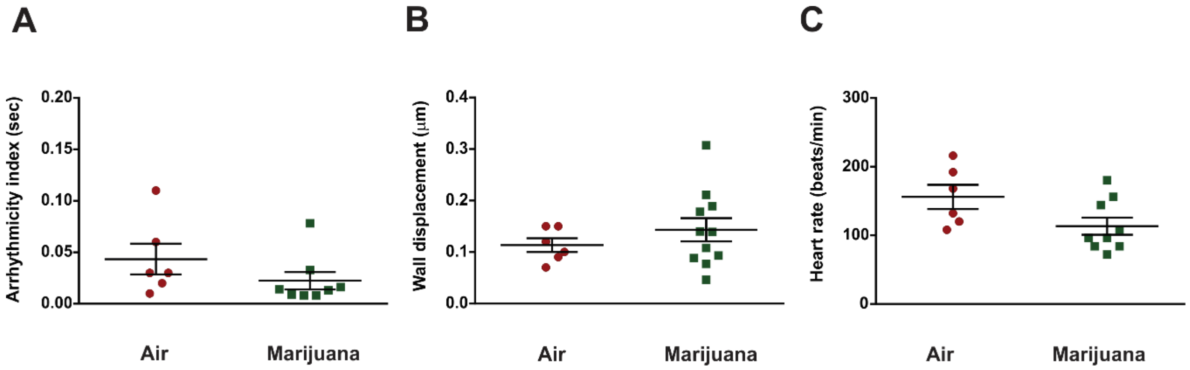

Figure S1. Treatment interruption corroborates that positive inotropic effects are induced by long exposure. 10 days old flies were subjected during the first 5 days to ambient air or cannabis and grown for another 5 days after treatment interruption. A-C: Average results (± SEM). A. Arrhythmicity index was not augmented in the treated group. N = 6, 8. B. Contractility is not augmented after treatment interruption. N = 6, 9. C. Heart rate is similar between both groups. N = 6,11. Student´s test (two tails) was utilized for comparison between two groups. A p-value < 0.05 was considered significant.

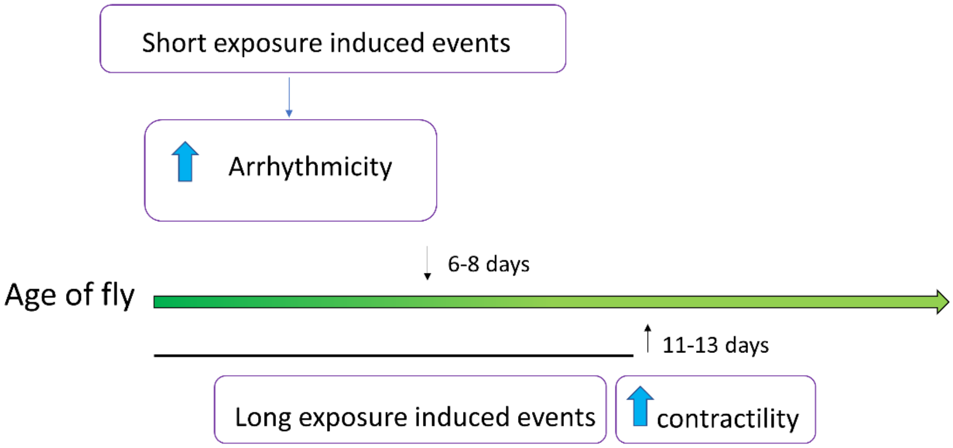

Figure S2. Classification of cardiac parameters affected by cannabis in relation to time of treatment and age of flies. Arrhythmic pattern is induced by short periods of treatment. Contractility is augmented only in response to long-term treatment.

Biology Open • Supplementary information

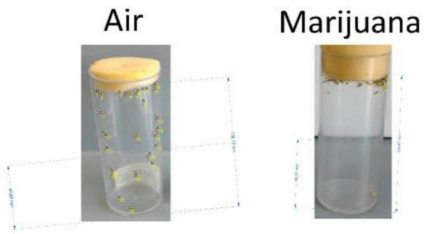

Percentage	Air	Marijuana
Top	75	88*
Bottom	25	12*

Figure S3. Flies exposed to cannabis exhibit a different behavioral pattern. Images captured from recordings of flies exposed to air or marijuana. Compared to controls (Air) flies receiving marijuana crowed at the top of the vials. Results are expressed as percentage of flies localizing to the top of the vials/total number of flies and represent the average of 12 images for treated and non-treated groups. Percentage comparisons were made using Fisher test. A p-value < 0.05 was considered significant.
